# Regulations against Cattle-tuberculosis in Switzerland

**Published:** 1898-01

**Authors:** 


REGULATIONS AGAINST CATTLE-TUBERCULOSIS IN SWITZERLAND.—On July 24, 1896, the Bundesrath decided to introduce tuberculin as a diagnostic means of exterminating tuberculosis. Further regulations relating to the same have lately been enacted. Tuberculin is to be distributed to the canton authorities free of charge. In order to guard against injurious changes in the tuberculin itself the orders for it must be commensurate with the actual need, and not sent in too long beforehand. The tuberculin is delivered ready for use in doses of three, four, and five cubic centimetres. In the orders it must be expressly stated how many doses of each kind are desired.

On the part of the canton authorities the tuberculin is to be given only to *qualified* veterinarians, the latter being authorized to administer the same and to report as to the results obtained.

As soon as a cattle-owner desires inspection for his cattle, the same must be applied not to individual animals, but to all cattle over six months old that the farmer possesses. Every animal that in consequence of tuberculin injection shows a rise in temperature of 1.5° or over is to be declared tuberculous, and to be marked by a triangle on the tip of the right ear. Tongs for this purpose are furnished by the Agricultural Department.

The costs of this tuberculin vaccination is to be met by the various governments.

It is encouraging that the use of tuberculin as a diagnostic is being consistently developed in Switzerland. In Germany its use is still confined to certain commercial centres where large importations of cattle are received from abroad.—*Idem.*, Feb. 6, 1897.

